# Response of murine fibroblasts or human keratinocytes to micro- and nano-scale titanium particles: the permeability of particles across keratinocytes' monolayers

**DOI:** 10.1007/s00784-025-06492-1

**Published:** 2025-09-30

**Authors:** Catarina Pacheco, Redouane Messous, Rui P Moura, Andreia Almeida, Patrícia Silva, Bruno Sarmento, Hassan Bousbaa, Júlio C. M. Souza

**Affiliations:** 1 UNIPRO – Oral Pathology and Rehabilitation Research Unit, University Institute of Health Sciences (IUCS), CESPU, Gandra PRD, 4585-116 Portugal; 2 i3S - Institute for Research and Innovation in Health, University of Porto, Porto, 4200-393 Portugal; 3 INEB - Institute of Biomedical Engineering, University of Porto, Porto, 4200-393 Portugal; 4 University Institute of Health Sciences (IUCS), CESPU, Gandra PRD, 4585-116 Portugal; 5 Center for MicroElectromechanical Systems (CMEMS-UMINHO), University of Minho, Guimarães, 4800-058 Portugal; 6 LABBELS - Associate laboratory, University of Minho, Braga, 4710-057 Portugal

**Keywords:** Cytotoxicity, Oral mucosa, Nanoparticles, Permeability, Titanium

## Abstract

**Purpose:**

The aim of this study was to evaluate the cytocompatibility of micro- and nano-scale commercially pure Titanium (cpTi) particles in contact with fibroblasts and keratinocytes and the penetration of cpTi particles across keratinocytes' layers.

**Method:**

Commercially pure titanium (cp-Ti) particles with 50-nm or 1-µm size were chemically and morphologically characterized using a Field Emission Guns Electron Microscopy (FEGSEM), Scanning Transmission Electron Microscope (STEM), and Energy Dispersion Spectrometry (EDAX). Then, the cytotoxic profile of the particles was monitored in contact with murine L929 fibroblasts and TR146 keratinocytes for 1, 4, and 7 days. Further permeability assays were performed across a TR146 monolayer via Transwell^TM^ model.

**Results:**

Physicochemical characterization of cpTi nano-scale particles (cpTi NPs) revealed a mean size at 70 nm and a specific surface area at around ~ 17.2 m^2^/g, while micro-scale particles (cpTi MP) size ranged from 0.3 up to 5.3 μm with a mean size of 1.4 μm at dry conditions. The optimized de-agglomeration of nanoparticles resulted in an increased specific surface area up to 57.3 m^2^/g. The metabolic activity of fibroblasts decreased against 50 or 100 µg/ml cpTi over 3 days cell culture while keratinocytes were not affected. Moreover, cpTi NP were internalized and steadily translocated into keratinocyte monolayers, showing an apparent permeability coefficient of 6.65 × 10^−6^ cm/s for 50 µg/mL and 3.96 × 10^−6^ cm/s for 100 µg/mL.

**Conclusions:**

Altogether, nano-scale titanium particles decreased the viability of fibroblasts although a significant viability of keratinocytes has been detected by standard cell culture assays. However, nano-scale titanium particles were found into keratinocytes and even trespassed the cells' layers that could reach other cells and blood vessels in an in vivo scenario. Thus, toxicity of titanium particles depends on their particle size, exposure time, content, and interaction with the surrounding media.

**Graphical Abstract:**

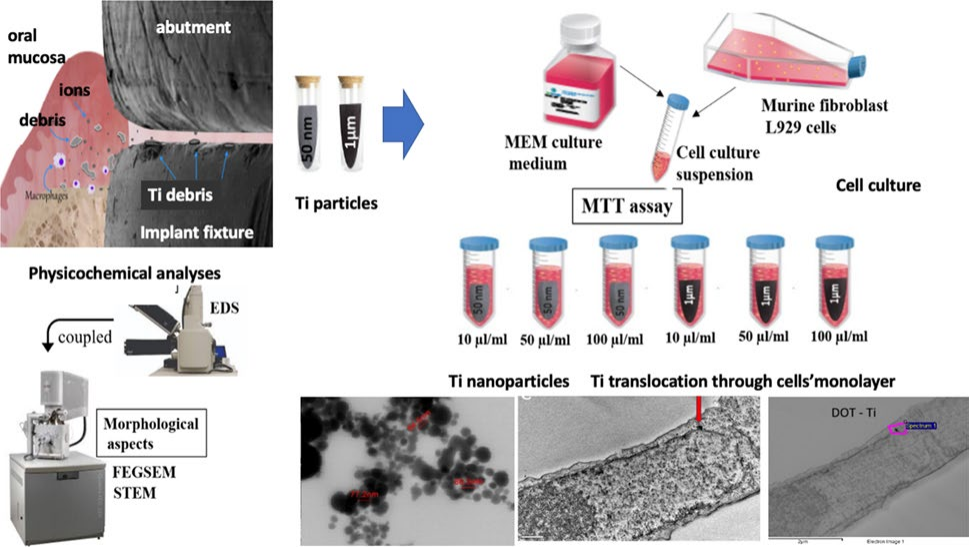

## Introduction

Although the success rates are high, 5–11% of dental implants fail within 10–15 years and must be removed [1–3]. The loss of a dental implant system is generally attributed to peri-implant inflammatory reactions leading to bone loss, namely peri-implantitis [1, 4, 5]. A complex co-aggregation of risk factors for implant failures takes place in most cases. Recently, a concern linked to the cyto- and genotoxic potential of metal ions and debris released from dental implant and abutment structures has been reported [6–10]. Ti, Al, and V ions and nano-scale particles (NPs) have been associated with peri-implant inflammatory reactions leading to osteolysis [11–14].

Titanium implant and abutment surfaces are coated with a thin layer of titanium oxide (i.e., TiO_2_, TiOH, Ti_2_O_3_) namely passive film, which forms spontaneously although that dynamically changes depending on the surrounding medium [11, 12, 15]. The chemical composition of titanium and its alloys is responsible for their corrosion resistance in different acidic media, especially in contact with saliva and gingival fluids [15, 16]. The passive film can become active when exposed to reactive substances such as lactic acid, citric acid, chlorides, and fluorides as found in the oral cavity [11, 12, 15–18]. The degradation of the titanium oxide film causes the release of Ti, Al, V ions as well as titanium-based debris at micro- and nano-scale size depending on the type of implant and abutment [16, 19]. In fact, cytotoxicity depends on the size, chemical composition, and content of metal ions and particles [15–19]. Metallic micro- (MPs) and nano-scale particles (NPs) released from dental implants as a result of corrosion and wear are considered foreign bodies that can activate the immune system [20–24]. Additionally, implant surfaces can be industrially nanostructured with different metallic or non-metallic elements and therefore such functionalized surfaces should provide a proper wear and corrosion resistance to the aggressive oral environment [19, 24, 25].

Thus, the translocation of metallic particles into the keratinocytes of the oral mucosa induce an indirect inflammatory response to the surrounding connective tissues and bone. Regarding the continuous presence of metal ions and MPs and NPs, the inflammatory response can be progressively occur since a strong osteoclastogenesis process takes place due to the activation of mature osteoclasts from macrophages’ responses [20–22]. Consequently, various inflammatory mediators and cytotoxins associated with peri-implantitis disease and bone resorption are activated by the surrounding cells [20–22, 26–33]. In an in vitro study, commercially pure titanium (cpTi) and TiO_2_ NPs induced higher cell internalization when compared to cpTi MPs when in contact with cells derived from the periodontal ligament [22]. Other studies reported the mutagenicity and genotoxicity induced by TiO_2_ NPs in different cell lines such as human osteoblast cells [13, 18, 30], rats’ fibroblasts [21, 34], human gingival fibroblasts [28, 35], oral epithelial cells [35–38], or macrophages [27, 32].

MPs and NPs released from titanium implants can accumulate in the surrounding tissues, or even spread systemically, as reported in previous studies contributing to the chronic inflammatory state in peri-implant disease [15–26, 36]. Thus, understanding the toxicity and the capability of the metallic particles to interact with the cells in close proximity is of paramount interest to clinicians to correctly assess the clinical risks. Peri-implant tissues comprises a stratified epithelium of the oral mucosa and the connective tissue. The oral epithelium acts as a barrier preventing xenobiotic penetration although NPs can breach such barrier towards the blood vessels leading to systemic exposure [35, 37–41]. At last, particles may accumulate in several organs such liver, kidneys, spleen, or lungs, causing toxic effects. For instance, studies have reported associations between Ti NPs systemic exposure and hepatic fibrosis, spleen injury, pulmonary vascular system thrombosis, and hepatocellular necrosis and apoptosis in mice [11, 12, 24].

The main objective of this work was to investigate the cytotoxic effect of micro- and nano-scale cpTi particles in contact with fibroblasts or keratinocytes followed by the particles' penetration assays through a monolayer of keratinocytes. A detailed physicochemical characterization of the Ti NPs was performed prior to the cell culture. Then, the particles' penetration was studied using an in vitro model of the oral mucosa. It has been hypothesized that high concentrations of Ti NPs should significantly decrease the viability of fibroblasts or keratinocytes and cpTi NPs could penetrate into oral epithelium cells.

## Materials and methods

### Preparation of cpTi stock suspension

Two groups of commercially pure titanium (cpTi) particles (SAT nano Technology Material CO., Ltd., China) with size at 1 μm or 50 nm (Product no. SAT-PWM200116) were assessed in this study. Stock solutions containing cpTi particles were prepared at a final concentration of 2 mg/mL (pH 4) in ultrapure water and 10% of fetal bovine serum (FBS; Gibco, USA) for the de-agglomeration of nano-particles. Particles were dispersed by sonication in the medium using an ultrasonic disintegrator (Vibra cell 75186, USA) equipped with a 19-mm Ti tip until no further advance in de-agglomeration process was achieved. Before setup the sonication, a calorimetric method was used to measure the specific energy as well as the sonication power transferred to the suspension. Sonication was then performed at 130 W of delivery acoustic power on pulse mode (pulse on for 60 s and pulse off for 10 s) and 50% amplitude in ice bath to avoid temperature rise for 8 min. The dispersion of the particles followed by physicochemical characterization was performed according to previous studies for comparison of findings [6, 17, 18, 42, 43]. Thus, several previous studies have utilized a similar pathway for particles’ dispersion concerning the protein corona effect. It should be emphasized the protein corona effect occurs spontaneous since the chemical interaction occurs between proteins and nano-scale particles in protein-rich medium, influencing further interactions in the surrounding environment [44–47].

### Physicochemical characterization of cpTi nano-scale particles

**Fig. 1:**
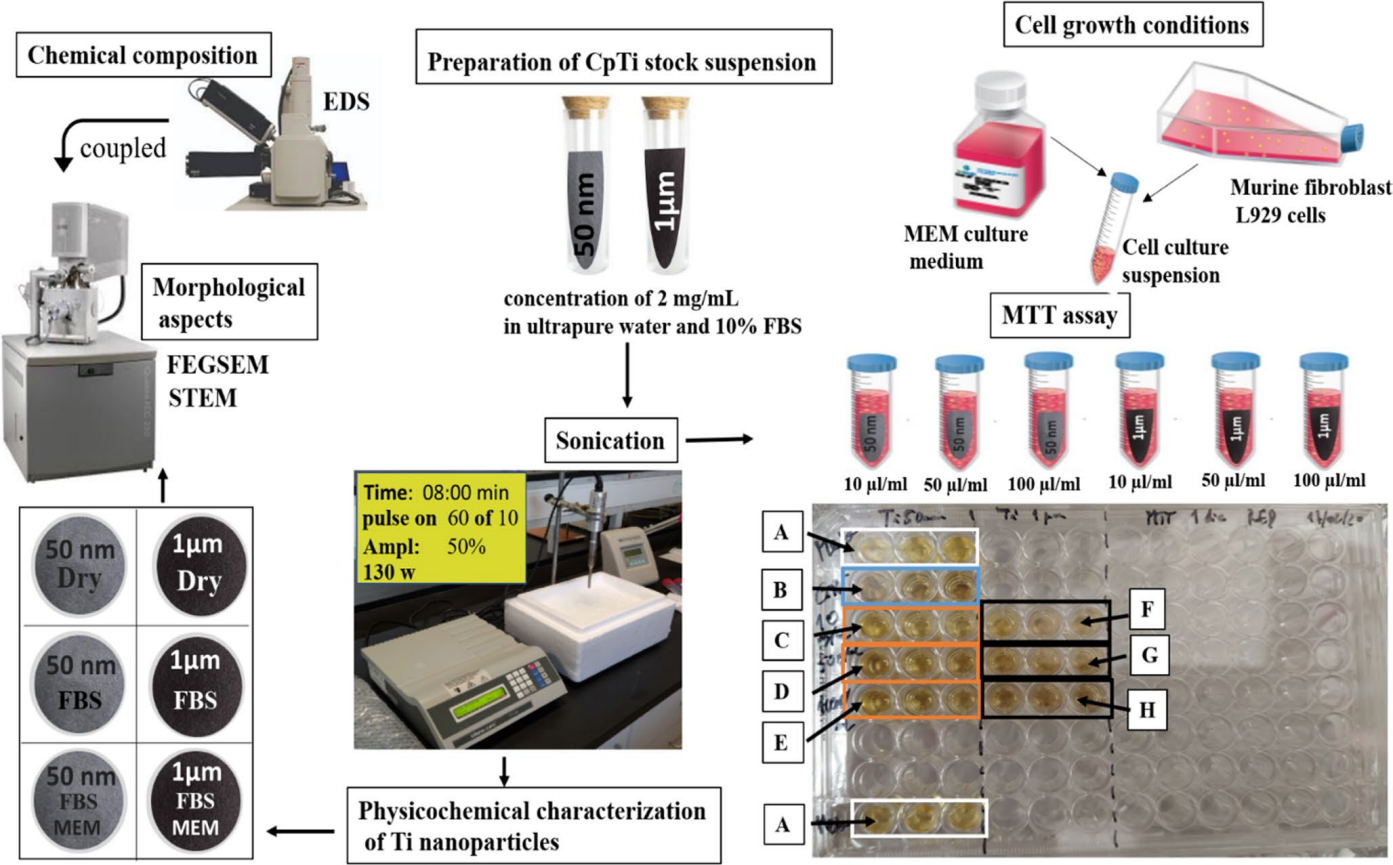
Schematics of the physicochemical characterization of cpTi NPs and cell viability assay. The setup of the 96-well flat bottom plates in which the fibroblasts cytotoxicity assay was carried out as follow: (**A**) culture medium free of cells and (**B**) including cells. Culture medium with cells in contact with cpTi particles with 50-nm size at (**C**) 10 µl/ml, (**D**) 50 µl/ml, or (**E**) 100 µl/ml. Culture medium with cells in contact with cpTi particles with 1-µm size at (**F**) 10 µl/ml, (**G**) 50 µl/ml, or (H) 100 µl/ml. Tests performed in triplicate

Six groups of samples were prepared for the physicochemical characterization of the cpTi particles in ultrapure water, FBS or FBS plus Modified Eagle’s Medium (MEM; Gibco, USA), as follow: (group A) CpTi powder particles with size at 50 nm or (group B) at 1 μm in dry conditions; (group C) CpTi powder particles with size at 50 nm or (group D) at 1 μm in ultrapure water; (group E) CpTi powder particles with size at 50 nm diluted in FBS or (group F) in a mixture of FBS and MEM solution; (group G) CpTi powder particles with size at 1 μm diluted in FBS or (group H) in a mixture of FBS and MEM solution (Fig. 1). The specific surface area of cpTi particle powder was determined by Brunauer-Emmet-Teller (BET) (Autosorb-1, Anton Paar QuantaTec Inc, USA). The volume of probe gas adsorbed was measured to determine the quantity of gas required to cover the dry particles’ surfaces.

Morphological aspects of the cpTi dry powder and particles’ suspension in culture medium were inspected using a Field Emission Guns Electron Microscopy (FEGSEM; FEI Nova 200, USA) and a Scanning Transmission Electron Microscope (STEM; FEI Nova 200, USA). The chemical composition of cpTi NPs was confirmed by Energy Dispersion Spectrometry (EDS) (EDAX; Pegasus x4M, USA) coupled to the FEGSEM. FEGSEM and TEM analyses were performed after cpTi particles stabilization in the culture media for 24 h. Then, suspension (10 µl) for gourps C-F were transferred onto glass discs with 12 mm in diameter. Analyses were performed in triplicate. Particles were analyzed after drying at room environment and then sputter-coating with a 2 nm-thin AuPd layer. For nano-scale particles, a drop of 50 µg/mL cpTi suspension was placed onto a holey carbon-coated copper grid, air-dried, and inspected by STEM.

Size distribution of cpTi particles was also investigated by dynamic light scattering (DLS) using a ZetaSizer Nano ZS (Malvern Instruments GmbH, Germany). DLS measurements were performed for particles in suspension (groups C-F) at 25 °C using standard 10 mm disposable optical polystyrene cuvettes. For each test condition, DLS measurements were performed in triplicate, with the test number and time for each run automatically determined by the apparatus’s software program. CpTi suspensions were homogenized by res-suspending the medium several times prior to filling the sample in the cuvette.

### Cell growth conditions

Murine fibroblast L929 cells (Laboratory of Stem Cells in Cancer and Aging, Univ. of Santiago de Compostela) were cultured in 25 cm^3^ culture flasks (VWR International, LLC, USA) containing MEM culture medium (Gibco, USA), and supplemented with 10% horse serum (HS), and maintained 37 °C in a 5% CO_2_ humidified incubator. After a 24 h incubation period, culture medium was discarded as cells washed with Hank’s Balanced Salt Solution (HBSS). Afterwards, cells were detached from the flasks using 0.025% trypsin (Sigma-Aldrich Co. LLC, USA) and harvested for posterior assays. Cell morphology was regularly monitored, and all experiments were performed with healthy exponentially growing L929 cells with more than 90% viability as determined by trypan blue exclusion assay. Also, human squamous carcinoma cells (TR146, Sigma-Aldrich, USA) were used at cell passage 37 to 39 for the cytocompatibility assays. For cell culture, Dulbecco’s Modified Eagle Medium (DMEM, Lonza, USA) was supplemented with 10% (v/v) fetal bovine serum (FBS) (Merck Millipore), 1% Penicillin/Streptomycin (Merck Millipore), and 1% (v/v) nonessential amino acids (NEAA, Merck Millipore). Both cells were seeded at a concentration of 1 × 10^4^ cells/cm^2^ for subcultivation once a week and media change was performed every 2–3 days.

### Cell viability assay

The cytotoxicity was assessed concerning the metabolic activity of L929 fibroblasts or TR146 according to the ISO 10993-5. Fibroblasts and keratinocytes were chosen considering cpTi debris are released by corrosion and wear surrounding the oral mucosa and connective tissues at the peri-implant region [11, 12, 16]. Cells were seeded in 96-well plates at a concentration of 5 × 10^4^ L929 or 1 × 10^4^ TR146 cells/well in contact with increasing concentration of particles. After the incubation period, cell metabolic activity of L929 or TR146 cells in direct contact with the cpTi particles was evaluated by colorimetric test using MTT [3-(4,5-dimethylthiazol-2-yl)−2,5-diphenyltetrazolium bromide] (Promega, USA), to assess the mitochondrial metabolic activity of the cells. MTT is a compound bio reduced by cells into a colored formazan product (purple color), which is soluble in HBSS. The number of metabolically active cells is directly proportional to the amount of formazan re-corded by spectrophotometry at 570 nm. The cell metabolic activity was analyzed as a function of time over a period of 1, 2, and 3 days of cell culture. The MTT assay results assume that the metabolic activity increase is due to the higher number of cells. For each period of cell culture, the culture medium and samples were removed and then the cells were washed three times with HBSS. Thereafter, 200 µL culture medium and 20 µL MTT reagent (1 mg/mL) were added in each well and the culture plates were incubated at 37 °C in an atmosphere of 5% CO_2_ for 4 h. Afterwards, 100 µL of solubilization solution (dimethyl sulfoxide) was added at each well to dissolve the formazan crystals followed by a 2 h incubation period. After being vigorously shaken for 10 min, absorbance of solubilized formazan crystals was measured by spectrophotometry at 570 and 630 nm in a microplate reader (Biotek, Synergy 2). The cells grown without any cpTi particles were used as positive control for metabolic activity of the cell line and all assays were performed in triplicate.

The percentage of cell viability was calculated using the following equation:

1 $$\begin{array}{lc}\%\;Cell\;viability\;=\\\frac{absorvance\;of\;treated\;cells\;-\;absorvance\;of\;negative\;control\;}{absorvance\;of\;untreated\;cells-absorvance\;of\;negative\;control}\\\times100\end{array}$$

### Permeability assays

For the permeability experiments, a monolayer of TR146 cells, from passage 37 to 39, was seeded on 12-well (3-μm pore size and 1.1cm^2^ of growth area) polystyrene cell culture inserts (Transwell™, Corning, Australia). Cells grown over a period of 21 days with a medium renewal every 48 h [35]. The permeability experiments were performed from the apical (0.5mL) to basolateral (1.5mL) compartment at 37 °C, using an orbital shaker (100 rpm). Before permeability experiments, medium was removed, cells were washed twice with HBSS at 37 °C, and incubated with HBSS, both in apical and basolateral compartment to equilibrate at 37 °C for 30 min. Then, 0.5mL cpTi NPs dispersions at concentrations of 50 µg/mL and 100 µg/mL were added into apical side of the inserts. At different time points (15, 30, 60, 120, 180, 240 min), 1.5mL were collected from basolateral compartment of the inserts and the same volume of pre-warmed HBSS was added to replace the harvested volume. Cell monolayer integrity was measured from the cells’ seeding until the last time point of the permeability experiment using an epithelial volt/Ohm meter (EVOM^2TM^, Thermofysher, USA). Transepithelial electrical resistance (TEER) values were expressed as percentage of TEER values in the beginning of the experiment.

The content of cpTi NPs permeated across the buccal cells were determined by atomic absorption spectroscopy using a graphite furnace as the atomizer chamber (AAS). Previously, calibration standards were prepared by a dilution of cpTi stock into 1% nitric acid. Then, 20µL cpTi standards or diluted samples in 1% nitric acid were injected into graphite tube. The parameters for the experiments were set by the AAA manufacturer and then optimized AAA furnace conditions were established by acquiring the curves of the standard solutions. All the measurements were performed on three independent replicates and based on peak absorbance of cpTi at 364.3 nm. Permeability results were shown as permeability percentage and apparent permeability coefficient (Papp). Papp was calculated as followed:

$$Papp\;=\frac{\triangle Q}{A\times C_O\times\triangle t}$$

where ΔQ equals cpTi particles quantified in basolateral compartment, A is the insert surface area (1.1 cm^2^), C_0_ is the initial concentration added to inserts apical compartment (50 and 100 µg/mL), and Δt is the complete experiment time in seconds (14400 s).

Additionally, cpTi NPs internalization into buccal epithelium cells was investigated by TEM (JEOL JEM-1400 microscope, JEOL lt., Japan). Briefly, 1 × 10^4^ TR146 cell were seeded per well in a 96-well plate and incubated overnight at 37 °C, 5% CO_2_, to ensure a monolayer formation. Before exposure to cpTi NPs, medium was harvested, and cells were rinsed twice with HBSS. Then, 0.2mL suspensions of 50 µg/mL cpTi NPs with 50-nm size were added and incubated for 30 min. Sample preparation and TEM analysis was performed at the Institute for Research and Innovation in Health (i3S), University of Porto [35]. TEM images were digitally recorded using a CCD digital camera Orious 1100 W and EDS spectra were acquired for chemical analysis to detect Ti chemical element.

### Statistical analysis

The statistical analysis of the experimental data was carried out using GraphPad prism 6 software (GraphPad Software, San Diego, USA). Normality tests were performed on the assumption that there was no relationship or difference between the variables. The results are presented as mean ± SD and groups were compared by two-way analysis of variance (ANOVA) followed by Tukey’s for multiple comparison test or an unpaired t-test. It was considered a level of statistical significance (α) at 5% (*p* < 0.05).

## Results and discussion

### Physicochemical characterization

**Fig. 2:**
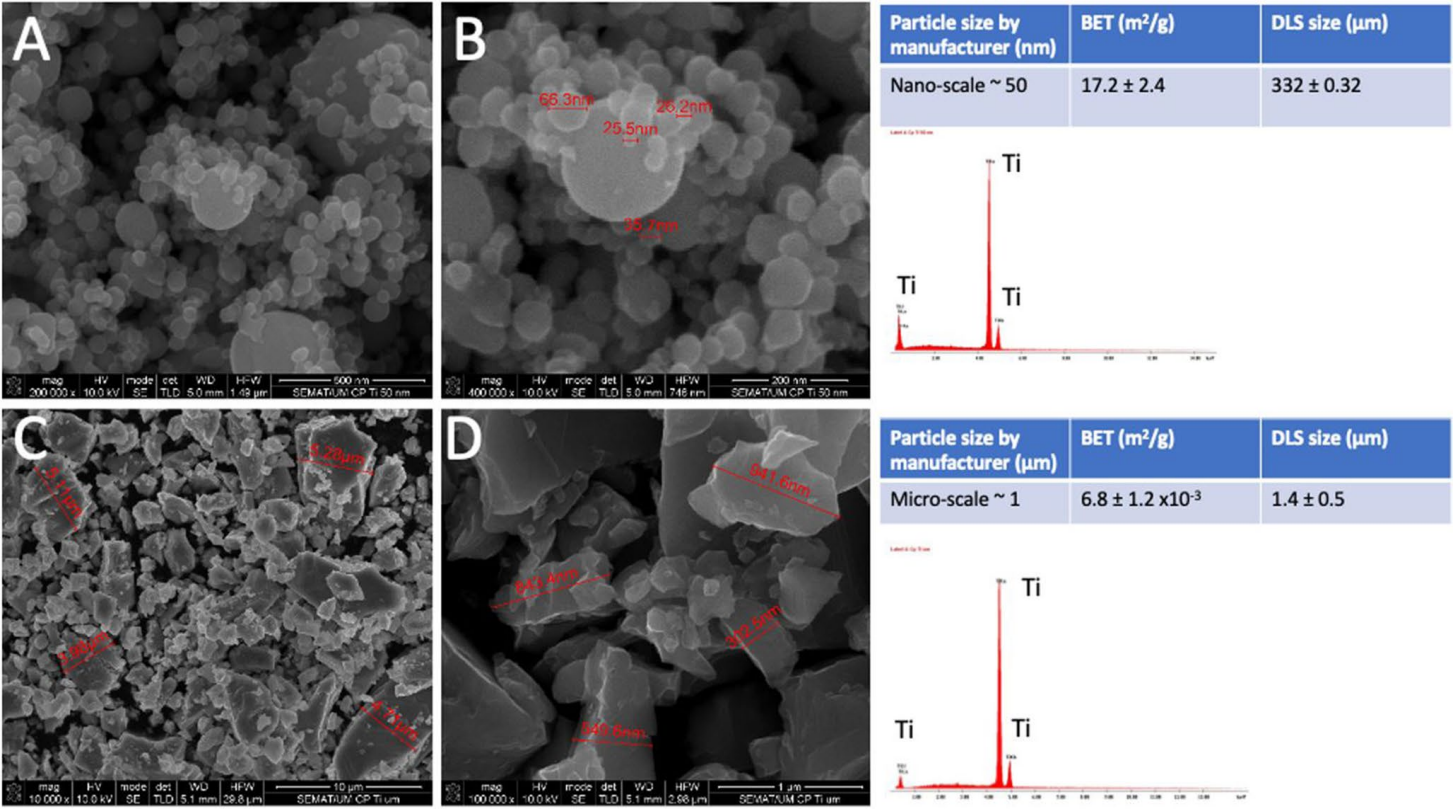
FESEM images of the cpTi (**A**, **B**) nano- and (**C**, **D**) micro-particles. FESEGM performed at 10 kV on secondary electrons (SE) mode. On the right side, elements maps recorded by EDS analyses

Physicochemical characterization of cpTi NPs by BET revealed a specific surface area of around ~ 17.2 m^2^/g, as seen in Fig. 2.

**Fig. 3:**
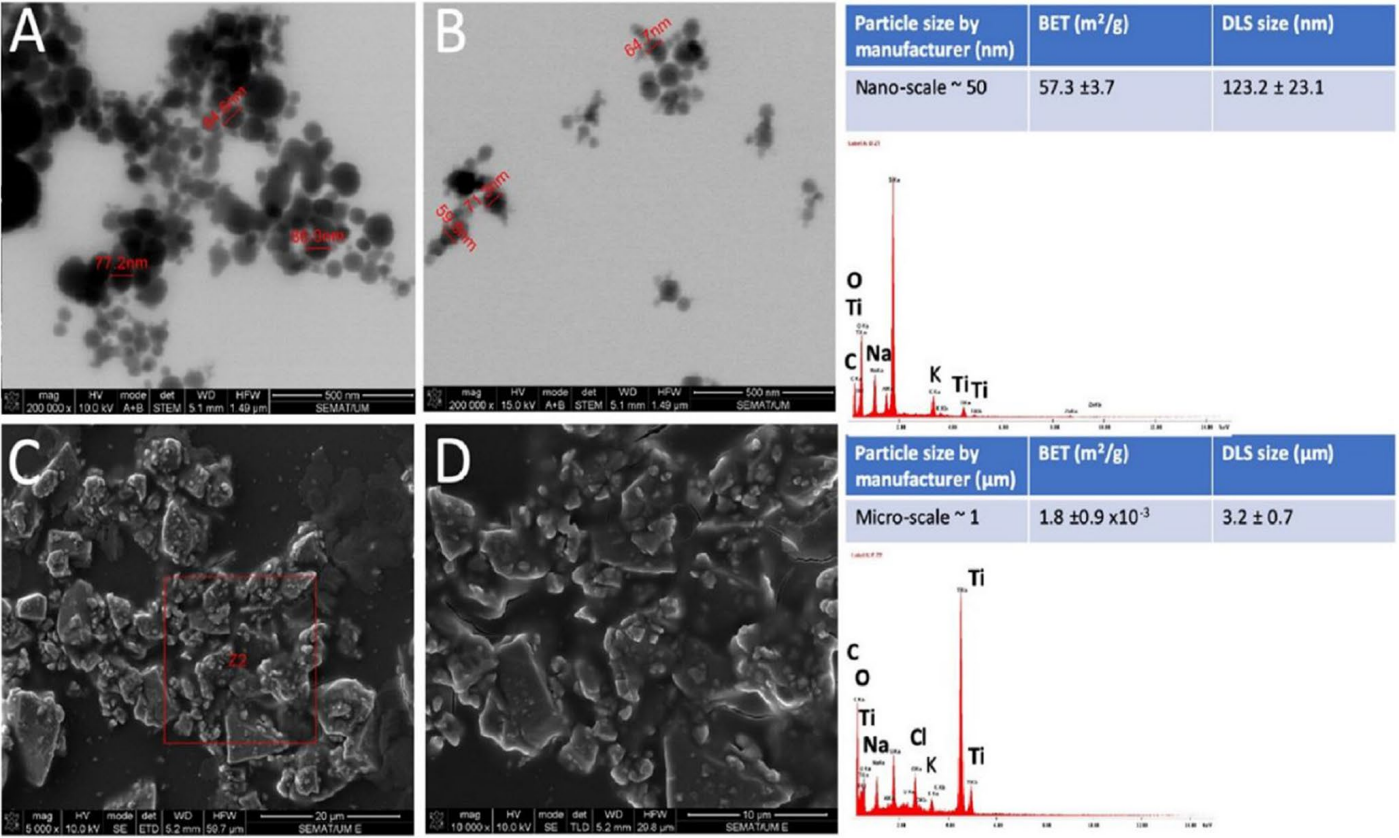
(**A**, **B**) STEM images of the cpTi NPs performed at 10 kV. (**C**, **D**) FEGSEM micro-particles performed on secondary electrons (SE) mode at 10 kV. On the right side, elements maps recorded by EDS analyses

Individual nano-scale particles at dry conditions were detected by FEGSEM and STEM at sizes ranging from 20 up to 90 nm with a mean size of 70 nm although particle agglomerates could be noted ranging from 200 up to 400 nm (Fig. 2A and B). CpTi MPs revealed sizes ranging from 0.3 up to 5.3 μm with a mean size of 1.4 μm at dry conditions, while particle agglomerates were noted at around 3 μm after interaction with the culture medium (Fig. 3C and D).

In this study, the sonication procedure of the particles prior to cell culture provided the de-agglomeration of the particles as noticed by the microscopic characterization. The optimized de-agglomeration of the nano-scale particles resulted in an increase of the individual nanoparticles proportion at around 60 nm leading to an increase in the specific surface area up to 57.3 m^2^/g as seen in Fig. 2. However, remnant particle agglomerates were still detected by FEGSEM and STEM that corroborated the DLS results. High-resolution TEM images demonstrated that protein from FBS medium surrounded individual particles and aggregates since FBS was used as a stabilization agent (Fig. 3).

Energy-dispersive X-ray spectra (EDS) elemental maps recorded on the cpTi particles are shown in Fig. 3. In fact, the particles established interaction with the compounds deriving from the culture medium (MEM) as indicated by the presence of (Na) sodium, (K) potassium, and (Cl) chloride (Fig. 2). The interaction of particles and salts occurs due to the chemical reactivity of the cpTi particles to the medium since K^+^, Na^+^, Ca^+2^, and PO^−^ form a stable layer on the cpTi surfaces. In FBS-rich medium, proteins are adsorbed on the stable layers as represented by C or N, that results in a bio-complex with a cpTi core that can influence the perception and interaction of the cells in the culture medium [42, 43, 45–49].

In a previous study, the size of cpTi NPs assessed was around 100 nm although cpTi particles/agglomerates with size higher than 100 nm were detected by SEM [27]. Physicochemical characterization of TiO_2_ indicated that their specific surface area was ~ 61.14 m^2^/g and that the individual particles were approximately 25 nm in size. The optimized de-agglomeration of the nano-scale particles resulted in an increase in the specific surface area with a mean hydrodynamic size of 142.1 ±5.6 nm due to the formation of a bio-complex composed of calcium (Ca), phosphorous (P) and proteins (e.g. albumin) deriving from the medium culture [18]. On the other hand, studies reported no agglomeration of cpTi micro-particles since the size and morphological aspects of the particles are maintained in culture medium [26, 28]. Thus, the agglomeration of cpTi nano-scale particles occurs, and the de-agglomeration must be performed prior to the culture medium to avoid bias of results. Since most of the studies have evaluated the effect of titanium oxide (TiO_2_) nano-scale particles, the agglomeration and de-agglomeration processes have been reported prior to the culture medium [6–10, 18]. There is a lack of information on the morphological aspects, chemical composition, and size of cpTi NPs regarding the agglomeration and de-agglomeration phenomena [22, 29]. However, the agglomeration of submicron particles (0.2–1 μm) has been inspected by FEGSEM and STEM in the literature [15, 16, 30–32].

### Biological assays

**Fig. 4:**
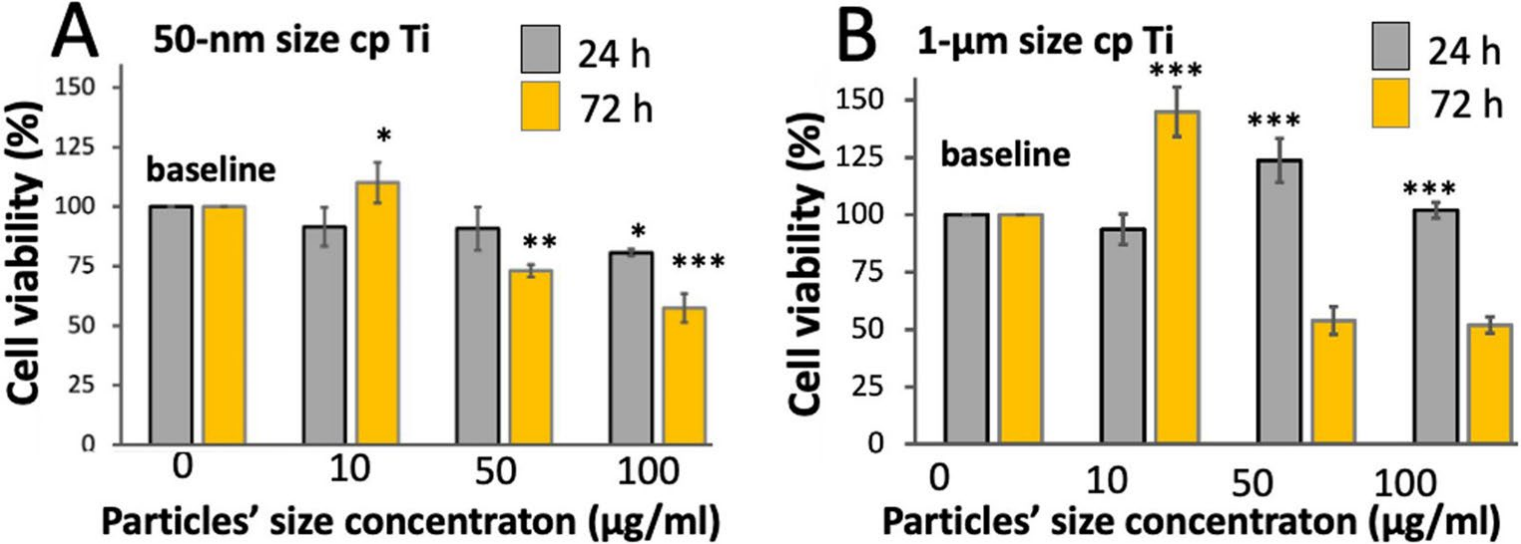
L929 fibroblast cell viability by MTT after exposure to cpTi (**A**) NPs and (**B**) MPs. Cell viability was assessed by MTT assay after 24, 48, or 72 h of exposure to cpTi particles with size at (**A**) 50 nm or (B) 1 μm and suspensions at concentrations ranging from 0.1 to 100 µg/mL. * *p* ≤ 0.05; ** *p* ≤ 0.005; ****; *p* ≤ 0.0001. Results are expressed as average of three replicates ± SD

Considering the effect of cpTi particles on cell viability, two different sizes (50 nm and 1 μm) and contents (10, 50, and 100 µg/mL) of cpTi particles were assessed in contact with L929 murine fibroblasts by MTT assays over three different time points (Fig. 4). The results partially validated the hypotheses since the 50-nm cpTi particles at 50, and 100 µg/mL decreased the proliferation of fibroblasts.

The principle of MTT test consists of the reduction of tetrazolium salts to formazan via mitochondrial reductase (Fig. 4). Regarding fibroblast viability after exposure to 50-nm particle size (Fig. 4A), a concentration of 10 µg/ml caused a lower decrease in the optical density of approximately (8.57%) when compared to 50 µg/ml cpTi NPs (9.28%) and 100 µg/ml (20%) for 1 day of incubation. On the incubation for 3 days, the decrease in cell viability was significantly higher at 100 µg/ml cpTi NPs (43%) when compared with 50 µg/ml (17%) although 10 µg/ml cpTi NPs did not affect the cell behavior (*p* ≤ 0.0001). Concerning cpTi particles with 1-µm size (Fig. 4B), a slight decrease in cell viability by (6.4%) was recorded at 10 µg/ml particles although no effect was noted at 50 µg/ml (Fig. 4). Therefore, an increase in cell viability was noticed at 100 µg/ml of cpTi particles (*p* < 0.05). On the incubation for 3 days, a decrease in cell viability by 46% was recorded at 50 µg/ml and by 48% at 100 µg/ml cpTi micro-particles (*p* < 0.0001).

In the culture medium, the minerals and proteins interacted with the MPs and NPs’ surfaces which disturbed the cell’s perception of the titanium particles. CpTi particles could effortlessly be internalized into the fibroblast considering the nano-scale size of the particles leading to genotoxic effects on the cells. Considering in vivo events, MPs and NPs are released and immediately interact with proteins, water, and minerals leading to a spontaneously functionalization of the particles. In fact, a kind of “Trojan-horse” effect takes places once the hybrid particles’ complexes are internalized by the surrounding cells [18, 42, 43, 45–49]. The importance the MPs and NPs properties in regulating biological responses have been recognized since the first findings on the protein corona effect [42, 43, 45–49]. In literature, different media are assessed such as blood, blood serum, plasma or complex protein mixtures. Also, other factors involving time, particles’ size & concentration, and temperature bring different results on the physicochemical pathways regarding the correlation of NPs’ properties and corona composition over time [42, 43, 45–49].

To the best of our knowledge, the effect of cpTi NPs has not been studied in contact with fibroblasts [21, 22]. In a previous in vitro study, metallic ions released from stainless steel, nickel-free alloys, and titanium orthodontic alloys were placed in the cultures of human fibroblasts to evaluate cell viability and DNA damage. MTT test results showed that the cell viability of fibroblasts in culture with Ti ions was higher when compared to stainless steel and nickel-free alloys [33]. However, the toxic effect of cpTi particles was reported against other cell lines such as monocytes and macrophages [16, 27, 32]. An in vitro study reported cpTi MPs < 20-μm size in culture of human monocytes (THP-1 cells) over a period of 12, 24, and 48 h [16]. MTS assays showed that titanium microparticles did not affect the metabolism of cells for 48 h of incubation, but RT-PCR demonstrated that cpTi MPs induced a proinflammatory response in macrophages characterized by increased expression of TNF-α and IL-6 cytokines known to stimulate bone resorption [16, 32].

The toxic effect of 5-nm TiO_2_ nano-scale particles at different content (from 3 to 600 µg/mL) against murine fibroblast L929 was reported by a previous study [34]. The MTT test indicated that cell viability was markedly reduced as the culture time and the concentration of TiO_2_ nano-scale increased [34]. Another previous study reported the negative osteoblast response when in contact with 25-nm TiO_2_ [18]. The bio-complex formed on TiO_2_ nano-scale particles was composed of calcium, phosphorous and proteins (albumin, glycoprotein-ALB protein and Alpha 2HS) deriving from the medium culture. Such bio-complexes’ layer within minerals and proteins allowed the internalization of TiO_2_ nano-scale particles by osteoblast cells like a “Trojan-like” effect [18]. Previous findings suggested that TiO_2_ NPs induced effects on osteoblasts are dependent on time and concentration. Morphological analysis of cell organelles indicated an adverse response, and DNA analysis showed extensive fragmentation that may disturb gene expression and cause mutagenesis in cell replication [7–10, 18].

Regarding cpTi MPs, a previous in vitro study revealed the cytotoxicity of cpTi particles of ≤ 20-μm size at concentrations of 0.01 to 1.0 mg/ml in culture of human gingival fibroblasts (HGF-1) and human calvaria osteoblasts (HCO) at three time points for 1, 7, and 21 days [30]. MTT testing showed a decrease in metabolic activity only for 1-day incubation and at the highest cpTi concentrations. The long-term toxic effect was slightly noticeable, although human gingival fibroblasts secreted interleukin 6 over the entire cell culture incubation. Such interleukin stimulates the differentiation of macrophages and osteoblast precursor cells into mature and active osteoclasts [30]. In contrast, another in vitro study was performed to determine the proliferation rate of human fibroblast cells in the presence of cpTi MPs at 0.488-μm size for three time points (1, 3, and 7 days) [31]. MTT results showed that inhibition of metabolic activity and cell viability decreased progressively over time [31]. Another in vitro study demonstrated that Ti6Al4V MPs (20 μm size) are also cytotoxic to Rat2 fibroblasts and MTT results showed a correlation between cytotoxicity and particle concentrations [12]. It was also shown that Ti6Al4V particles at 20 μm size induced cytotoxic effects in human osteoblast cultures. MTT assays were performed at four time points 1, 2, 3 and 7 days at concentrations of 100, 50, 10, 1, 0.5, and 0.1 µg/mL. According to the results, cell viability decreased at high concentrations of Ti MPs [13].

**Fig. 5:**
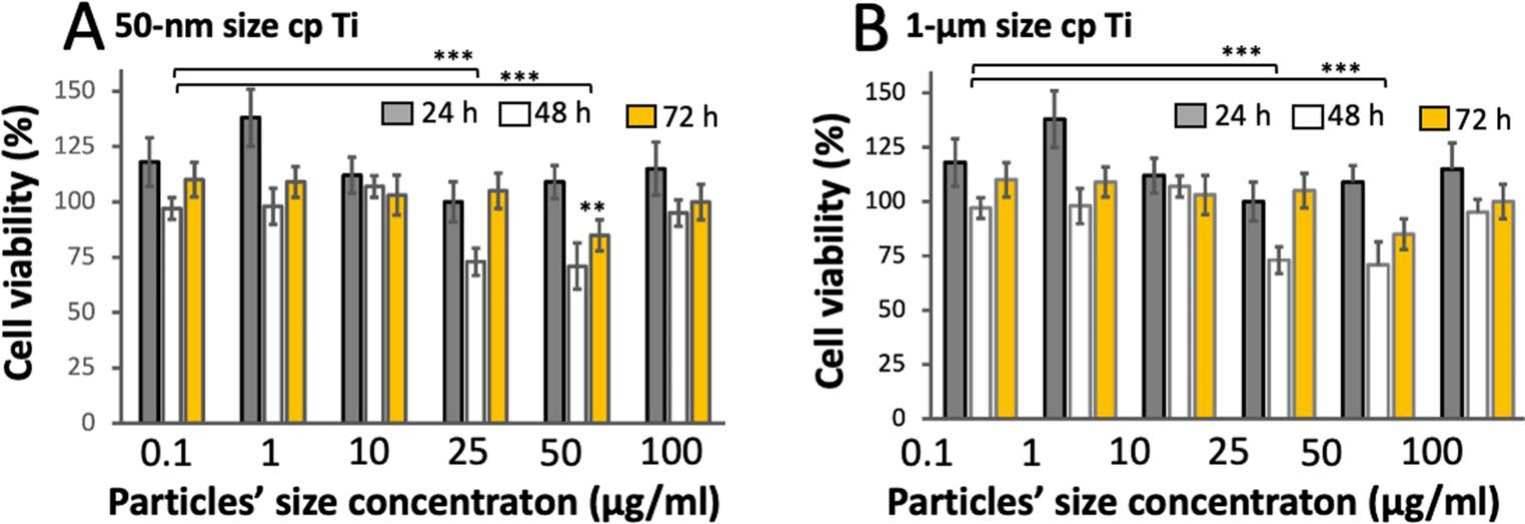
TR146 cell viability after exposure to cpTi (**A**) NPs and (**B**) MPs. Cell viability was assessed by MTT assay after 24, 48, or 72 h of exposure to cpTi particles with size at (**A**) 50 nm or (B) 1 μm and suspensions at concentrations ranging from 0.1 to 100 µg/mL. Results are expressed as average of five replicates ± SD. * *p* ≤ 0.05; ** *p* ≤ 0.005; ****; *p* ≤ 0.0001. Results are expressed as average of three replicates ± SD

 In this study, the in vitro cytotoxicity of the cpTi MPs and NPs was also evaluated in contact with TR146 keratinocytes by MTT assay, as seen in Fig. 5. Cell viability results showed a decrease in the cell viability at MPs and NPs concentration around 25 and 50 µg/mL for 48 h cell culture. As seen in Fig. 5, there was low a variation in cell viability within the tested concentration ranges for each particle size although the cell viability remained higher than 70% under all experimental conditions.

**Fig. 6:**
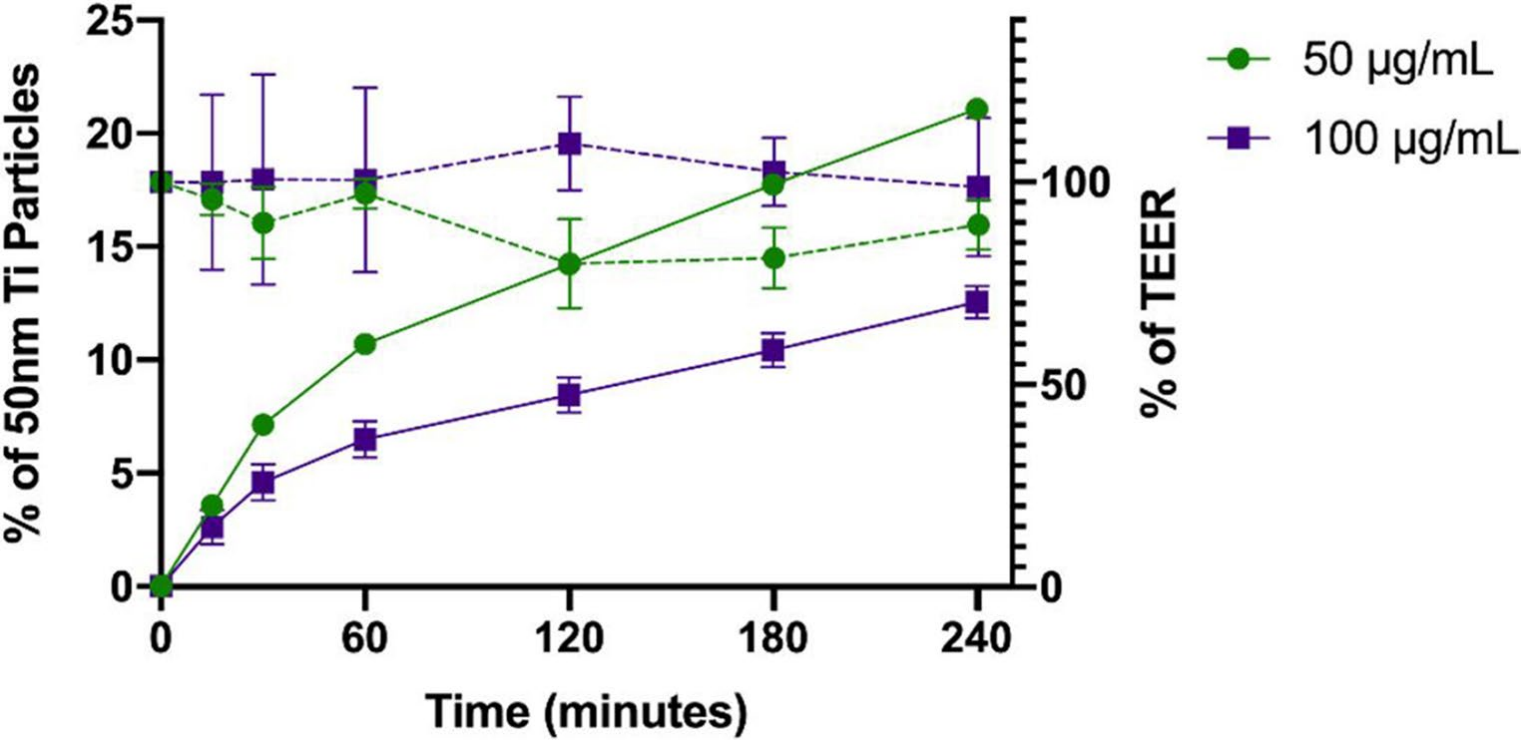
Percentage of TR146 transepithelial electrical resistance (TEER) (dashed line) and in vitro cumulative permeability profile (full line) of 50 nm cpTi NPs across a TR146 cell monolayer at 50–100 µg/mL over 240 min. The experiments were conducted at 37 °C from the apical to basolateral compartment in HBSS. Results are expressed as average of three replicates ± SD

The permeability of 50-nm cpTi particles (50–100 µg/mL) across buccal epithelial cells using TR146 cells' monolayers via the Transwell™ model (HTS Transwell™, Corning, Norway) was studied and the results are shown in Fig. 6. Following 21 days of culture in well-plates, TR146 keratinocytes formed an intact monolayer exhibiting morphological and functional characteristics similar to normal human oral mucosa. Transepithelial electrical resistance of TR146 monolayers (TEER) was monitored for 21 days and, all monolayers presented TEER mean values above 400 Ω × cm^2^, which indicates its integrity, ideal to perform permeability assays [41, 50]. Transwell™ with TR146 cells is a simple, easily reproducible, and consistent in vitro model, often used to screen compound permeability through oral epithelium [35, 39]. Even though TR146 are human squamous carcinoma cells, the morphological and grown on in vitro conditions are proper for permeability assays of metallic cpTi MPs and NPs. Thus, TR146 cell culture in Transwell™ is a valuable and useful in vitro model despite its tendency to underestimate compound permeability and to not faithfully recapitulate the oral mucosa mucous layer [38, 40, 51–53].

As seen in Figs. 6 and 50-nm cpTi particle permeability reached ~ 21% at 50 µg/mL and ~ 12% at 100 µg/mL for 240 min.

Results indicate particle permeability across the TR146 monolayer is not dependent on the concentration to which cells are exposed. Additionally, cpTi particles permeability occurred steadily throughout the experiment with high TEER values. The TEER is an indicator of the monolayer integrity and barrier properties and therefore, probably, 50-nm cpTi particles translocate the buccal cells via a saturable transcellular transport. Further studies are required to verify the nature of nano-scale particles interaction and transport through the cells. Clathrin-mediated or caveolin-mediated endocytosis may be involved, as those proteins have been reportedly participating in the uptake of nano-scale particles by endocytic vesicular membrane within similar structures (38,46).

The apparent permeability coefficient (Papp) found for 50-nm cpTi particles through the TR146 monolayer was recorded at 6.65 × 10^−6^cm/s for 50 µg/mL and at 3.96 × 10^−6^cm/s for 100 µg/mL content. Such values indicate a high permeability of Ti particles across buccal cells.

**Fig. 7:**
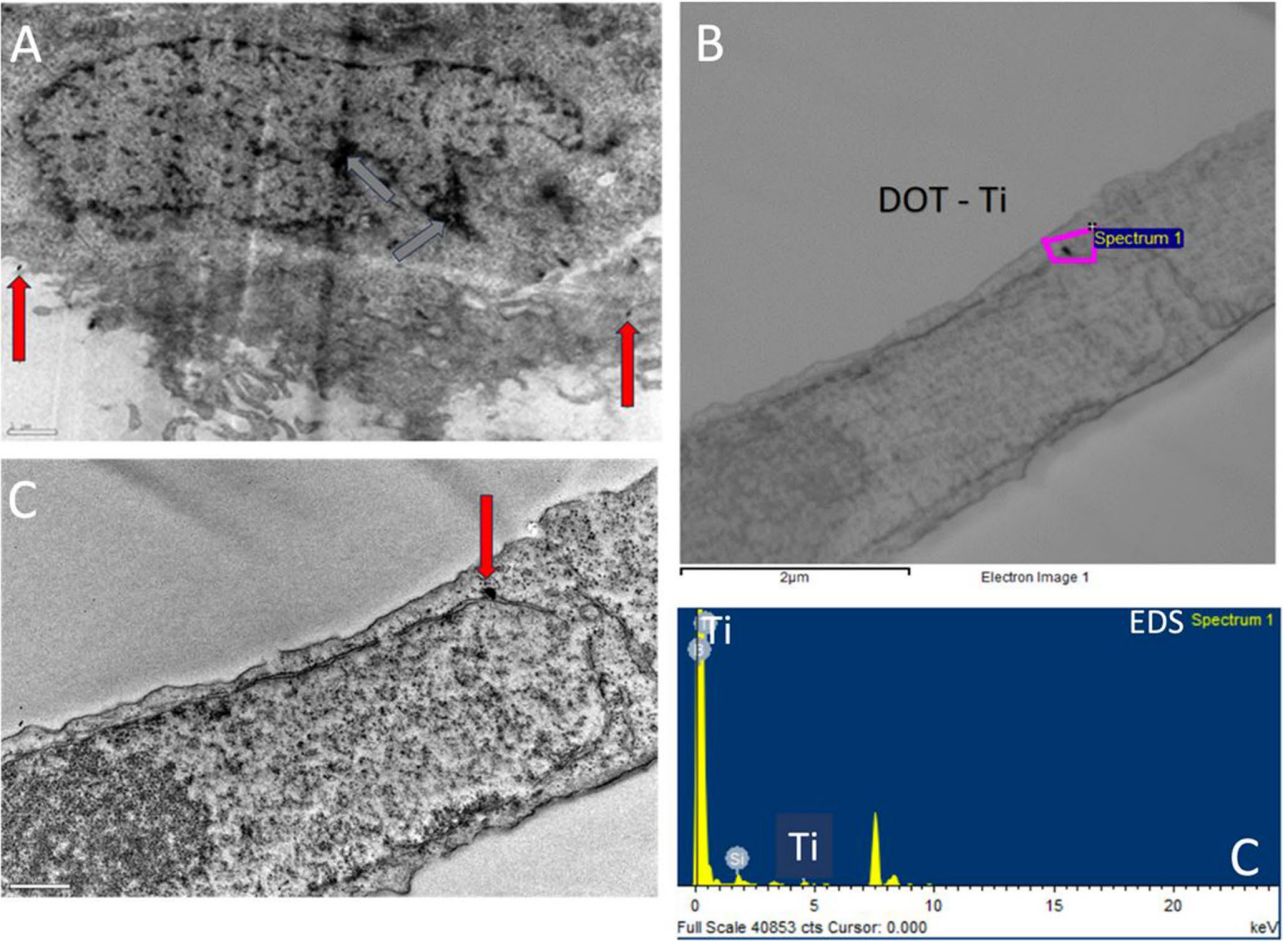
Uptake of 50 nm cpTi NPs by TR146 cells for 30 min. (**A**) Representative TEM micrographs of TR146 cells incubated with 50 µg/mL of cpTi NPs. Red arrows indicate the presence of cpTi NPs while grey arrows indicate cellular structures where Ti presence was not detected. Top image, scale bar: 1 μm. Bottom image, scale bar: 0.5 μm. (**B**) TEM micrograph showing a selected area for (**C**) EDS analysis and corresponding element mapping (bottom), confirming the presence of Ti element

TEM results on TR146 cells after incubation for 30 min (Fig. 7) clearly showed cpTi NPs internalization by the cells and their distribution throughout the cytoplasm, although NPs outside the cells were also detected. Those findings align with data recorded from the permeability assays, in which nano-scale particles permeability through an intact TR146 monolayer is higher than 5% after 30 min incubation. Our findings are not the only ones demonstrating cpTi NPs internalization through the oral epithelium. A previous study has also reported smaller Ti NPs (~ 24 nm) internalization and accumulation into TR146 monolayers [35]. Also, other studies have shown that Ti NPs penetrate the mucosal layer and subsequently the oral epithelium in fresh porcine tissue, with a noticeable distribution in the cellular cytoplasm [37]. Simultaneously, similar findings have been reported for other regions of the gastrointestinal and respiratory tracts [39]. Considering the internalization and chemical reactivity of cpTi NPs, it should be highlighted that higher concentrations of titanium NPs may cause damage to oral epithelial cells, namely DNA alterations.

In vitro studies involving dispersion of NPs have several limitations concerning the properties of NPs and the composition of the protein corona. In this study, physicochemical analyses of titanium MPs and NPs were accomplished to provide data on the chemical composition of the particles’ surfaces before and after interaction with cell culture medium. Then, the cell viability was assessed in contact with titanium MPs and NPs in cell culture with fibroblasts and keratinocytes. In the same way, further studies should focus on different types and size of particles released from prosthetic and implant materials and therefore cell culture studies with different cell lines are required in function of the cell culture medium and time. The influence of metallic NPs and protein corona composition must be clarified concerning the biological activity, cell uptake, biodistribution, and drug delivery in the surrounding environment. The toxicity of titanium MPs and NPs should be reconsidered in light of the protein corona effects and therefore novel methods on proteomics and machine learning can be combined on the analyses of the protein corona composition and effects.

## Conclusions

Within the in vitro limitations of a physicochemical analysis and cytocompatibility of titanium micro- and nano-scale particles, concluding remarks can be drawn as follow.

Concerning cpTi particles with 1-µm size, a decrease in fibroblasts metabolic activity was recorded for concentrations of 50–100 µg/ml over 3 days. Nano-scale particles of titanium with size ranging around 50–70 nm also induced marked decrease in L929 fibroblast viability at concentrations of 100 µg/ml over a period of 3 days. A decrease in the keratinocytes’ viability was noticed at concentrations of 25–50 µg/ml of cpTi nano-scale particles for 48 h although cpTi particles with 1-µm size did not reveal any toxic effects to keratinocytes. At 50–100 µg/ml content, cpTi nano-scale particles penetrated keratinocytes and steadily translocated intact keratinocytes monolayers. Nevertheless, this study showed limitations on the in vitro de-agglomeration procedure of the cpTi nano-scale particles and assessment of different cells taking into account the correlation between particles’ size and cell viability. Future studies are required to clarify the toxic and cells’ uptake mechanisms of the cpTi nano- and submicron particles on the buccal cells and tissues surrounding the implant. The presence of protein corona and bioactive salts composed of calcium and phosphates should be correlated with the internalization pathways of nano-scale particles into the cells. Exploratory studies could also assess the impact of cpTi particles on macrophages, osteoblasts, mesenchymal cells, and on the presence of inflammatory mediators and cytokines such as toll-like receptor 2 ligands.

## Data Availability

No datasets were generated or analysed during the current study.
